# Two cases of extramedullary plasmacytoma of the small intestine presenting with ileus: A case report

**DOI:** 10.1097/MD.0000000000030842

**Published:** 2022-10-07

**Authors:** Ki Bum Park, Hyung Jun Kwon, Ji Yun Jeong, Dong Won Baek, Seung Hyun Cho, An Na Seo

**Affiliations:** a Department of Surgery, School of Medicine, Kyungpook National University, Kyungpook National University Chilgok Hospital, Daegu, South Korea; b Department of Pathology, School of Medicine, Kyungpook National University, Kyungpook National University Chilgok Hospital, Daegu, South Korea; c Department of Internal Medicine, School of Medicine, Kyungpook National University, Kyungpook National University Chilgok Hospital, Daegu, South Korea; d Department of Radiology, School of Medicine, Kyungpook National University, Kyungpook National University Chilgok Hospital, Daegu, South Korea.

**Keywords:** extramedullary plasmacytoma, ileus, multiple myeloma, small intestine

## Abstract

Extramedullary (extraosseous) plasmacytoma (EMP) of the small intestine is a rare plasma cell neoplasm (PCN) that presents with abdominal pain, nausea, vomiting, etc. We describe 2 cases of EMP of the small intestine that presented with ileus.

**Methods:**

A 78-year-old woman and 68-year-old man visited our clinic with complaints of abdominal discomfort and obstruction. Abdominal computed tomography (CT) revealed a thickened lesion with multiple enlarged mesenteric lymph nodes (LNs) in the ileum and duodenum. The female patient underwent segmental resection in the ileum. The male patient underwent Whipple’s operation in the duodenum.

**Results:**

Histopathological examination and immunohistochemical staining of resected specimens from the 2 patients confirmed a PCN. In the surgical specimens of 2 cases, immunoglobulin heavy-chain rearrangement was confirmed by polymerase chain reaction amplification, but no Epstein-Barr virus (EBV)-infected cells were found by EBV-in situ hybridization. Bone marrow aspirate and trephine biopsies were negative for the type of PCN. Bone marrow cytogenetics and fluorescence in situ hybridization revealed no abnormalities. Serum β2-microglobulin levels were within normal limits. Additionally, none of the patients showed an M-spike in serum or urine protein electrophoresis. Therefore, the patients were diagnosed with a solitary EMP of the small intestine. The female patient refused treatment. At follow-up 3 months postoperatively, her disease progressed and she newly developed multiple LNs and nodular lesions in the right pelvic side wall. She was treated with dexamethasone. The male patient experienced back pain 25 days after Whipple’s operation. Spine series magnetic resonance imaging revealed an intermediate signal intensity mass in the posterior epidural space from T8/9 to T10. The mass was removed, and the same histologic features were identified as duodenal masses. He was treated with dexamethasone and radiotherapy.

**Conclusions:**

EMPs of the small intestine are easy to overlook because they rarely occur in the small intestine. Although surgery is not required for diagnosis, surgical resection can be a good option for EMPs of the small intestine, instead of local radiation therapy. However, close follow-up is required due to the possibility of relapse or progression to plasma cell myeloma.

## 1. Introduction

Extramedullary (extraosseous) plasmacytoma (EMP) is a localized tumor consisting of monoclonal plasma cells that arise in tissues other than the bone. Plasmacytoma presents no clinical features of plasma cell myeloma (PCM) and is classified into 2 types: solitary plasmacytoma of the bone (SPB) and EMP. The incidence of EMP accounts for approximately 1% of plasma cell neoplasms (PCNs) and rarely involves the gastrointestinal tract (GI).^[1,2]^ Rodrigo et al reported a comprehensive review of the published literature of 61 cases presenting EMP of the small intestine through a literature search.^[2]^ Herein, we report 2 cases of EMP in the ileum and duodenum in a 78-year-old woman and a 68-year-old man who presented with small bowel obstruction.

## 2. Case report

This study was approved by the Institutional Review Board of Kyungpook National University Chilgok Hospital (KNUCH), and the requirement for informed consent was waived given the de-identification of all patients’ personal information.

### 2.1. Case 1

**Figure 1.**
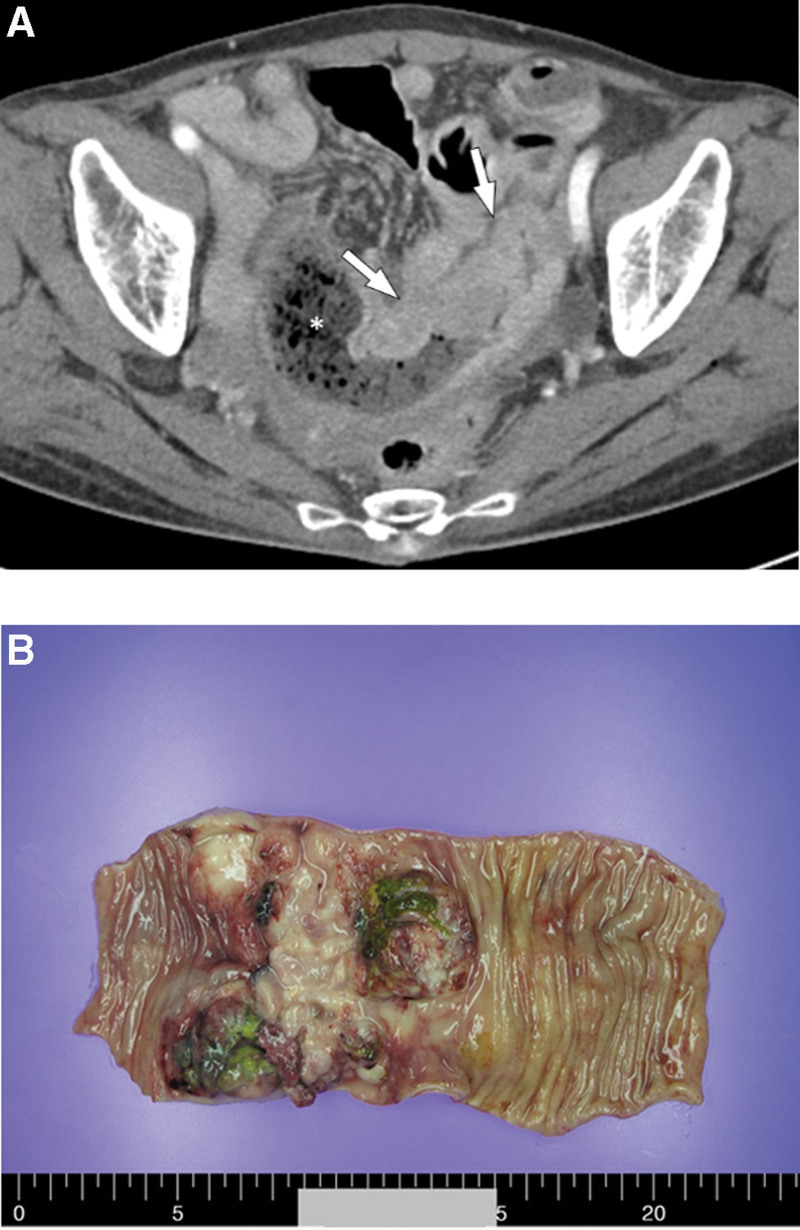
(A) A computed tomography scan of the abdomen and pelvis shows 7-cm long segmental enhancing wall thickening of the ileum with obstructive ileus and multiple enlarged mesenteric lymph nodes. (B) The surgical resected specimen shows a large ulcero fungating mass measuring 10.0 × 8.0 cm.

**Figure 2.**
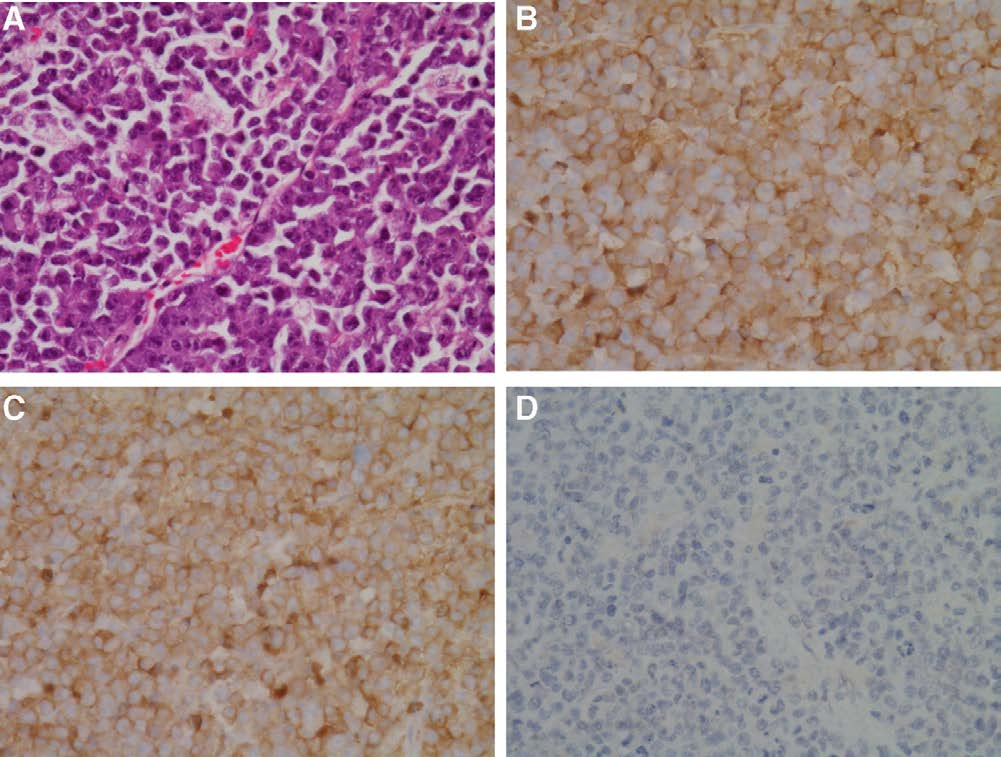
(A) Hematoxylin and eosin staining shows abnormal plasma cell infiltrate in the ileum (×40 objective). Immunohistochemically, these atypical plasma cells stained positively for (B) kappa light chains and (C) lambda light chains, but not for CD20 (×40 objective).

A 78-year-old woman visited the emergency room with nausea and vomiting for 10 days on July 31, 2021. She had a history of being diagnosed with peripheral T-cell lymphoma occurring in her left lower neck in 1999 and was completely cured. At the time of admission, abdominal and pelvic computed tomography (CT) showed 7-cm long segmental enhancing wall thickening of the ileum with obstructive ileus of the upstream small bowel loops and multiple enlarged mesenteric lymph nodes (LNs) (Fig. 1A). Based on these radiological features, a clinical diagnosis of small bowel lymphoma was suspected. She did not exhibit any symptoms of anemia, hypercalcemia, or renal insufficiency. No abnormal findings were observed in the preliminary laboratory tests performed before surgery. The patient underwent an exploratory laparotomy. Operative findings showed a small bowel mass in the ileum with proximal small bowel edema. We performed segmental resection and anastomosis of the small bowel on August 16, 2021. A drain tube was inserted near the anastomosis site. The nasogastric tube was removed immediately after surgery. A clear-water diet was started on postoperative day 3. The diet was changed gradually from clear water to a liquid and soft diet. The patient was discharged 7 days after surgery without complications. Gross examination of the resected small bowel showed a smooth serosal surface and a large ulcerating mass measuring 10.0 × 8.0 cm in the ileum (Fig. 1B). On sectioning, the tumor was yellow, with well-demarcated boundaries invading the serosal layer. Histological examination of the surgically resected specimen showed infiltration of atypical plasma cells with relatively uniform and round-to-oval eccentric nuclei, nuclear membrane irregularity, infrequent prominent nucleoli, and dispersed nuclear chromatin (Fig. 2A). The tumor invaded the serosa, and 1 of the 6 regional LNs was involved in the neoplasm. Immunohistochemically, almost all of the tumor cells stained strongly and diffusely for plasma cell markers, lambda, kappa, and MUM-1, but the results for CD20, CD79a, PAX5, CD138, BCL6, and cytokeratin staining were negative (Fig. 2B–D). In addition, the Ki-67 proliferative index was approximately 90%. Clonal immunoglobulin heavy-chain (IGH) rearrangement (IdentiClone® IGH Gene Clonality Assay, InVivoScribe Technologies, CA) was performed using formalin-fixed paraffin-embedded tissue, and monoclonality was identified. By contrast, Epstein-Barr virus (EBV)-infected cells were not found in formalin-fixed paraffin-embedded tissues using EBV in situ hybridization (Ventana® EBER ISH iView Blue Plus Kit, Ventana Medical Systems, Inc., AZ).

After the identification of the neoplastic proliferation of plasma cells, further tests were performed. No specific findings were observed in the serum and urine protein electrophoresis and immunofixation. Serum and urine immunoelectrophoresis did not reveal monoclonal gammopathy. However, serum free kappa light chains presented 31.07 mg/L (normal limit, 3.30–19.40 mg/L), whereas free lambda light chains showed 327.86 m/L (normal limit, 5.71–26.30 mg/L). The kappa/lambda ratio was 0.09 (normal limit, 0.26–1.65). Quantitative immunoglobulin (Ig) levels were normal (IgG, 833 mg/dL; IgA, 216 mg/dL; and IgM, 70 mg/dL). Additionally, bone marrow aspirate and trephine biopsies were negative. Bone marrow cytogenetics and fluorescence in situ hybridization revealed no abnormalities. Therefore, PCM was excluded and a final diagnosis of EMP of the small intestine was made.

Positron emission tomography/CT performed 1 month after surgery showed hypermetabolic LNs in the left paratracheal station and inferior mesenteric area. With progressive disease, chemotherapy was recommended, but the patient refused treatment owing to old age and poor general condition. At follow-up 3 months after surgery, her disease progressed and she newly developed multiple LNs and nodular lesions in the right pelvic side wall. She was treated with dexamethasone and is still alive.

### 2.2. Case 2

**Figure 3.**
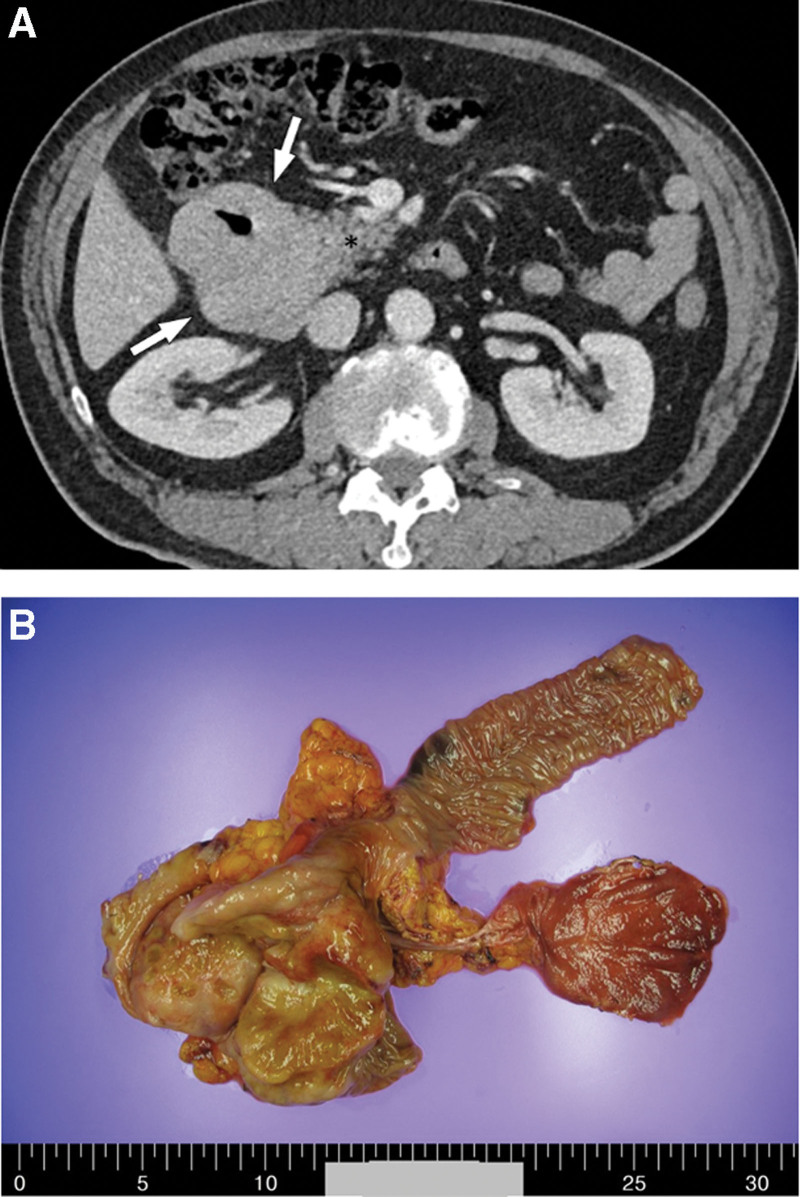
(A) A computed tomography scan of the abdomen and pelvis shows wall thickening with ulcer in the duodenum and multiple lymphadenopathy. (B) The surgical resected specimen shows a large ulcero fungating mass measuring 10.5 × 7.5 cm.

**Figure 4.**
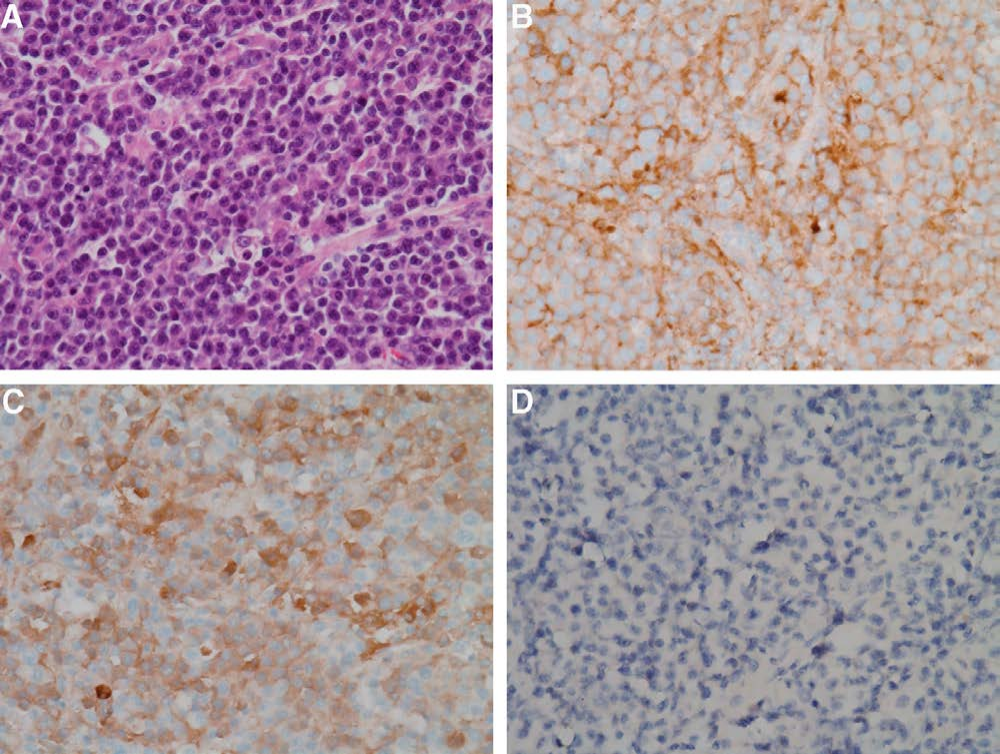
(A) Histology of the tumor shows atypical plasma cell infiltration in the Hematoxylin and eosin staining. These abnormal plasma cells stained positively for (B) kappa light chains and (C) lambda light chains, but not for CD20 (×40 objective).

**Figure 5.**
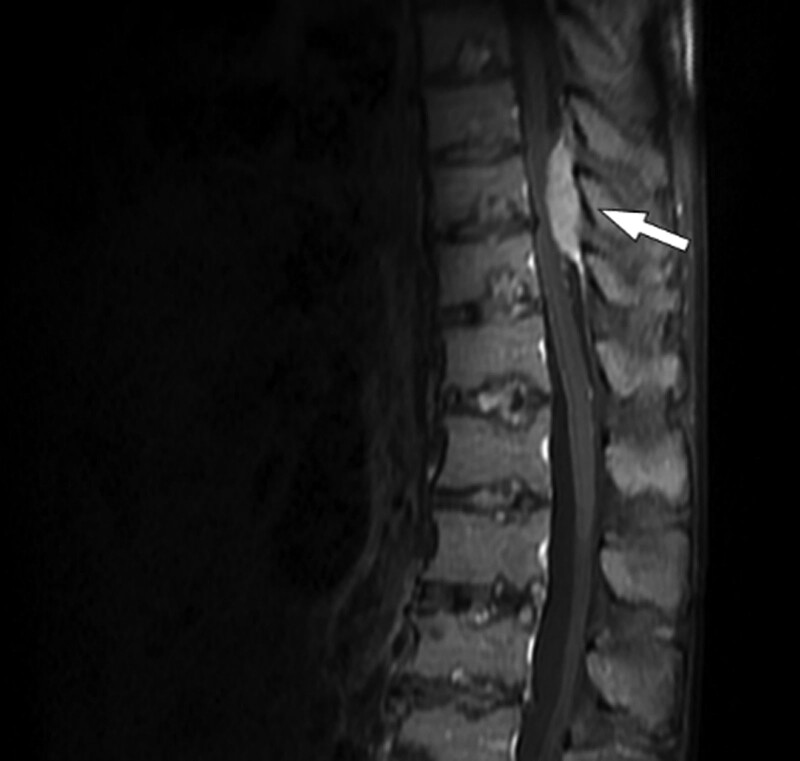
Spinal magnetic resonance imaging shows a mass in the posterior epidural space from T8/9 to T10 upper body level.

A 68-year-old man presented to our hospital with melena and abdominal discomfort in September 2020. He visited another local hospital and was treated for a suspected duodenal ulcer on esophagogastroduodenoscopy. However, despite treatment, his symptoms did not improve, and he was admitted to the gastroenterology department in October 2021. Abdominal and pelvic CT revealed wall thickening with an ulcer in the duodenum; multiple lymphadenopathies were also observed (Fig. 3A). Preliminary laboratory tests were normal, except for anemia (hemoglobin concentration, 8.5 g/dL; hematocrit, 28.4%). He did not show any symptoms of hypercalcemia or renal insufficiency. Because the clinical diagnosis was suspected to be duodenal adenocarcinoma, a biopsy via esophagogastroduodenoscopy was performed, but only ulcer detritus tissue was obtained in November 2021. Surgery was decided upon for diagnosis and treatment, and blood transfusion was performed promptly before surgery because the patient had melena for a long time. He underwent Whipple’s operation in December 2021. On gross examination, the duodenal mass measured 10.5 × 7.5 cm with ulceration (Fig. 3B). Histopathological examination showed diffuse infiltration of atypical plasma cells in the duodenal wall and mucosa and involvement of the ampulla of Vater, but not the pancreas, stomach, or distal common bile duct (Fig. 4A). The neoplastic nature of the plasma cell infiltrate was confirmed by immunohistochemical staining, which was positive for CD138, a plasma cell marker, kappa, lambda, and MUM-1, but negative for CD20 and CD79a expressions (Fig. 4B–D). The Ki-67 proliferative index was ~90%. In addition, the IGH rearrangement showed monoclonality, whereas EBV-infected cells were not identified. Twenty days after surgery, the patient complained of back pain, and a mass was found in the posterior epidural space from T8/9 to T10 upper body level on spinal magnetic resonance imaging Fig. 5). The mass was removed via laminectomy, and the histologic features of the mass in the T spine were consistent with those of the mass in the duodenum. To exclude extraosseous infiltrates of the PCM, additional examinations were performed. Bone marrow aspirate and trephine biopsies revealed no signs of PCM. Serum and urine protein electrophoresis did not yield a monoclonal spike in the gamma-globulin region. Serum and urine immunoelectrophoresis did not show monoclonal gammopathy. Furthermore, his anemia was identified as microcytic hypochromic anemia, which was suspected to be iron-deficiency anemia caused by melena for over 1 year. Because no other site was observed, except for the mass found on the T spine, we diagnosed EMP of the small intestine with metastasis in the posterior epidural space of the T spine. The patient was treated with dexamethasone and radiotherapy (40 Gy in 20fractions). Since then, he has been disease-free.

## 3. Discussion

EMP is a rare hematological malignancy that involves organs outside the bone, representing about 1% of PCNs.^[1,2]^ EMP show normal bone marrow with no evidence of clonal plasma cells in radiographical and morphological assessments.^[3]^ In addition, the diagnosis of EMP should have no clinical features of PCM. Infrequently, EMP can progress to develop PCM, occurring in about 15% of EMP.^[1,4]^ Two-thirds of patients with EMP are men, and the median age at diagnosis is about 55 years.^[2,5]^ Most EMPs are localized in the mucous membranes of the upper respiratory tract; more rarely, they can affect the GI tract, skin, bladder, LNs, and central nervous system.^[1]^ However, because of the occurrence of extraosseous sites, the differentiation from lymphomas that exhibit plasma cell differentiation (such as marginal zone lymphoma of the mucosa-associated lymphoid tissue type, lymphoplasmacytic lymphoma, and plasmablastic lymphoma) is necessary. In particular, distinction from marginal zone lymphoma with extensive plasma cell differentiation is difficult in the GI tract, skin, and upper respiratory tract, and it may be impossible by morphological assessment.^[6]^ Among the 2 cases, the female patient had a past history of complete cure of peripheral T-cell lymphoma onset in 1999 after being treated and had been monitored during regular follow-up. Therefore, lymphoma was the first clinically differentially diagnosed despite the uncommon symptoms of bowel obstruction on radiographic imaging. To improve the symptoms of bowel obstruction and confirm the diagnosis, she underwent emergency segmental resection of the small intestine. Although the resected specimens were detected with clonal IGH, CD20, CD79a, and PAX5 were all negative, whereas the plasma cell markers kappa and lambda showed strong and diffuse positive results in the immunohistochemical assessment. Based on the immunohistochemistry results, the diagnosis was considered PCN. The male patient showed similar molecular and immunohistochemical characteristics. As such, the distinction of EMP from lymphomas with extreme plasma cell differentiation is crucial because treatment and prognosis differ among them.^[2,7]^ For this, immunohistochemistry or immunophenotyping can help make a correct diagnosis. However, in some cases, EMP and lymphomas with marked plasma cell differentiation cannot be distinguished with certainty using immunohistochemistry alone. In such cases, molecular alterations may be helpful, although the molecular features of EMP have not been fully studied. The molecular features of EMP seem to be similar to those of PCM, except in the absence of t(11;14) translocations and *MYC* rearrangement.^[8,9]^ EBV infection is rarely found in EMP, but is detected in 50% to 70% of plasmablastic lymphomas.^[10]^

A comprehensive review of the 61 published cases of EMPs of the small intestine, that was conducted as a literature search of the databases of journals, was reported and patients’ characteristics and clinical manifestations were summarized.^[2]^ Of 61 published EMPs of the small intestine, 20 (32.8%) cases were found in the duodenum, 24 (39.3%) in the jejunum, and 17 (27.9%) in the ileum.^[2]^ Almost all of these cases are described as primary, although secondary EMP is more commonly frequent than primary EMP. ^[2]^ Among 41 EMPs of the jejunum and ileum, 40 cases are confined to these anatomic locations, but EMPs of the duodenum often involve the stomach.^[2,11]^ Their clinical presentations varied, since symptoms are related to the effect of the mass on the surrounding structures.^[2,12]^ Possible symptoms are abdominal pain, intestinal obstruction, intussusception, nausea, vomiting, constipation, weight loss, malabsorption, GI bleeding, and obstructive jaundice.^[2]^ Our female patient also had EMP of the ileum, which is a primary type, although she had a past history of lymphoma 22 years ago. Her symptoms and bowel obstruction may also have occurred because of the large mass, measuring 10.0 × 8.0 cm. Unlike our case, Alnimer et al reported a case of multiple EMPs of the ileum in a 51-year-old woman who presented with small bowel obstruction and had a previous history of solitary plasmacytoma of the left acetabulum.^[12]^ They described that their case developed multiple EMPs of the ileum 3 years after the diagnosis of SPB.^[12]^ By contrast, our male patient developed metastasis in the left posterior epidural space of T 8–10 spine due to late diagnosis of EMP. He was surgically diagnosed 1 year after experiencing melena symptoms. He also showed partial obstruction due to the large mass, measuring 10.5 × 7.5 cm. Fortunately, metastasis was observed at only 1 site, and after treatment, the patient is now fine.

As regards treatment, several retrospective studies have reported that the effective treatment for EMP is local radiotherapy.^[13,14]^ Especially, EMP is a highly radiosensitive tumor that can be controlled in 80% to 100% of cases with moderate doses (optimal radiation dose: 40–50 Gy in 20–25 fractions) of radiotherapy.^[14]^ However, the prompt surgical resection of the tumor could be another good alternative therapeutic option for GI EMPs, having the lowest recurrence rate.^[2,15]^ Furthermore, in the light of our 2 cases, it can be an option for accurate diagnosis. If the tumor can be completely resected, the long-term outcome is equal to that of patients receiving radiotherapy alone.^[1,2]^ However, if complete surgical resection of the tumor is impossible or doubtful and/or if LNs are involved, combined surgery and radiotherapy is recommended.^[1,15]^

EMP has a better prognosis than PCM and SPB, with about 70% of the patients remaining disease-free over 10 years.^[16]^ Regional relapse develops in approximately 25% of patients, and progression to PCM occurs in about 15%.^[1,4]^ In EMP of the small intestine, only 3 cases were reported to progress to PCM.^[17-19]^ Because the progression to PCM has a worse prognosis, regular follow-up is necessary using clinical, laboratory, and radiographic images.^[2,20]^

In summary, we describe 2 cases of EMP in the ileum and duodenum that presented with obstruction of the small intestine. These cases imply that EMP of the small intestine causes a diagnostic dilemma because of its rare clinical presentation and nonspecific symptoms. In addition, it is difficult to differentiate EMP from lymphoma using imaging studies and histological examination owing to its strong resemblance. Therefore, instead of local radiation therapy, surgical resection can be a good option for EMP of the small intestine for final diagnosis and decent treatment. Although EMP has a better prognosis than PCM and SPB, close follow-up is required due to the possibility of relapse or progression to PCM.

## Author contributions

**Conceptualization:** An Na Seo

**Data curation:** Ki Bum Park, Hyung Jun Kwon, Dong Won Baek, Seung Hyun Cho, An Na Seo.

**Formal analysis:** Ki Bum Park, An Na Seo.

**Funding acquisition:** Ki Bum Park

**Investigation:** Ki Bum Park, An Na Seo.

**Methodology:** Ki Bum Park, Hyung Jun Kwon, Ji Yun Jeong, Dong Won Baek, Seung Hyun Cho, and An Na Seo.

**Project administration:** An Na Seo.

**Writing – original draft:** Ki Bum Park, An Na Seo.

**Writing – review & editing:** Ki Bum Park, Hyung Jun Kwon, Ji Yun Jeong, Dong Won Baek, Seung Hyun Cho, and An Na Seo.
